# Supplementary figures and images for: ELISA with recombinant antigen Lb6H validated for the diagnosis of American tegumentary leishmaniasis
Source: PLoS One. 2024 Jun 5;19(6):e0304268. doi: 10.1371/journal.pone.0304268 (PMC11152253; doi:10.1371/journal.pone.0304268)

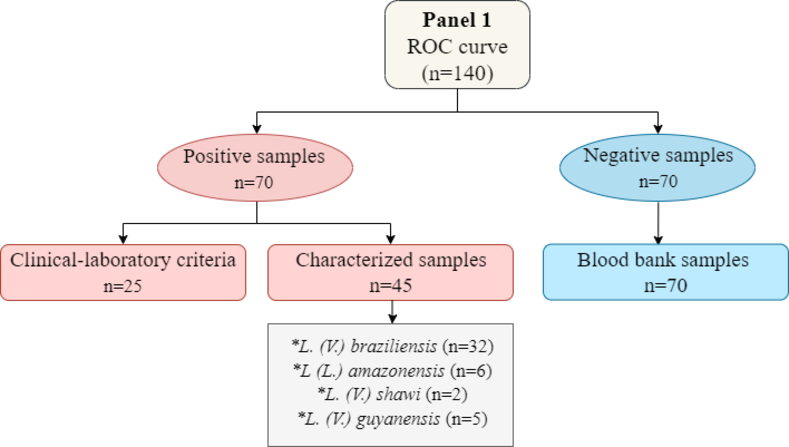

Supplement: S1 Fig — * Diagnosis based on clinical-laboratory criteria. **Diagnosis based on positive parasite culture and Leishmania species identification. n = number of samples. (TIF) [file pone.0304268.s002.tif]

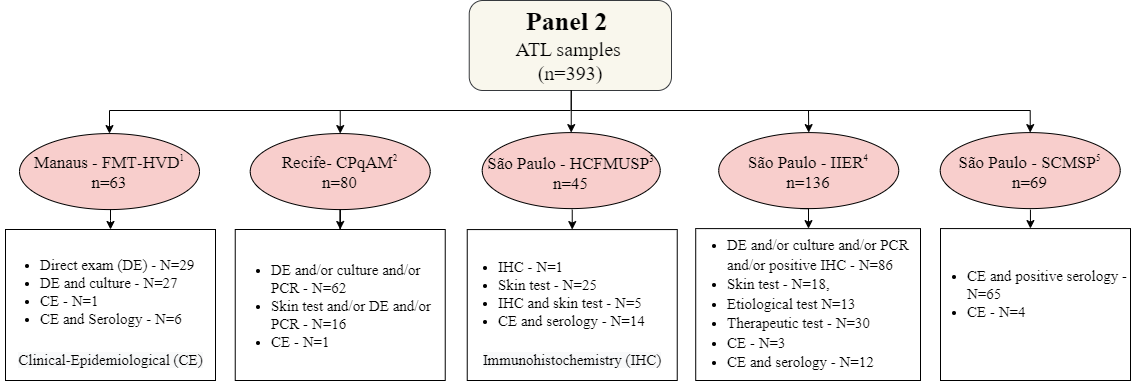

Supplement: S2 Fig — FMTHVD—Fundacao de Medicina Tropical Dr. Heitor Vieira Dourado, Manaus, AM; CPqAM -Centro de Pesquisas Aggeu Magalhaes, FIOCRUZ, Recife, PE; HCFMUSP—Hospital das Clínicas da Faculdade de Medicina da Universidade de Sao Paulo, Sao Paulo, SP; IIER—Instituto de Infectologia Emilio Ribas, Sao Paulo, SP; SCMSP–Santa Casa de Misericórdia de Sao Paulo, Sao Paulo, SP. n = number of samples. (TIF) [file pone.0304268.s003.tif]

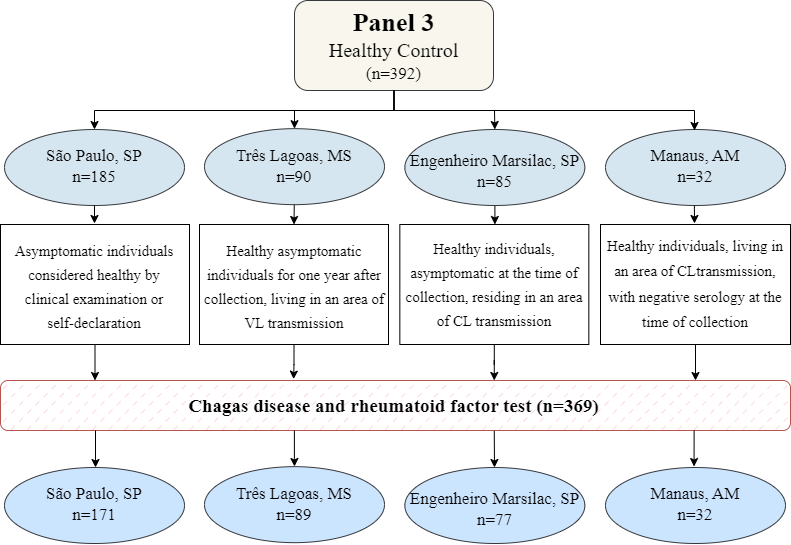

Supplement: S3 Fig — n = number of samples. (TIF) [file pone.0304268.s004.tif]

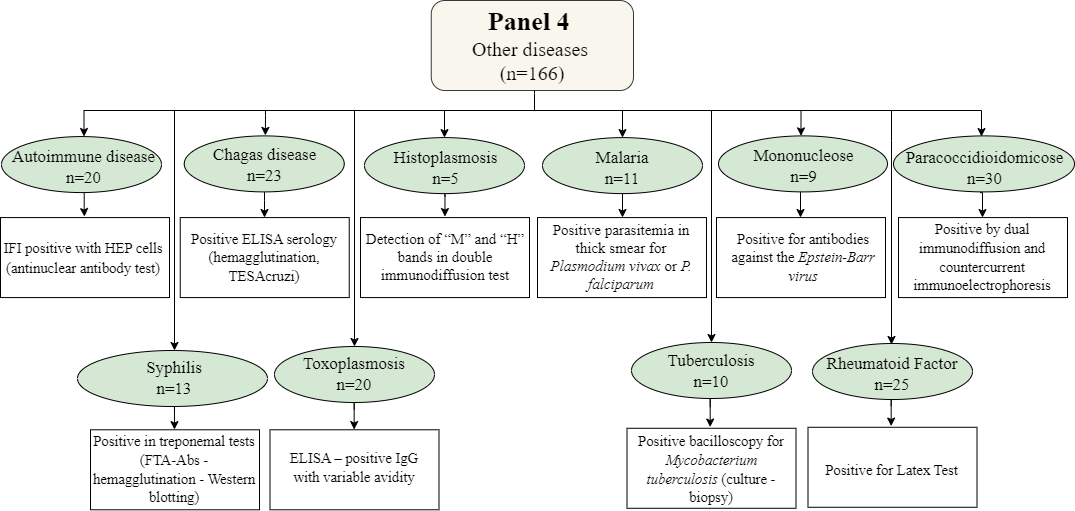

Supplement: S4 Fig — n = number of samples. (TIF) [file pone.0304268.s005.tif]

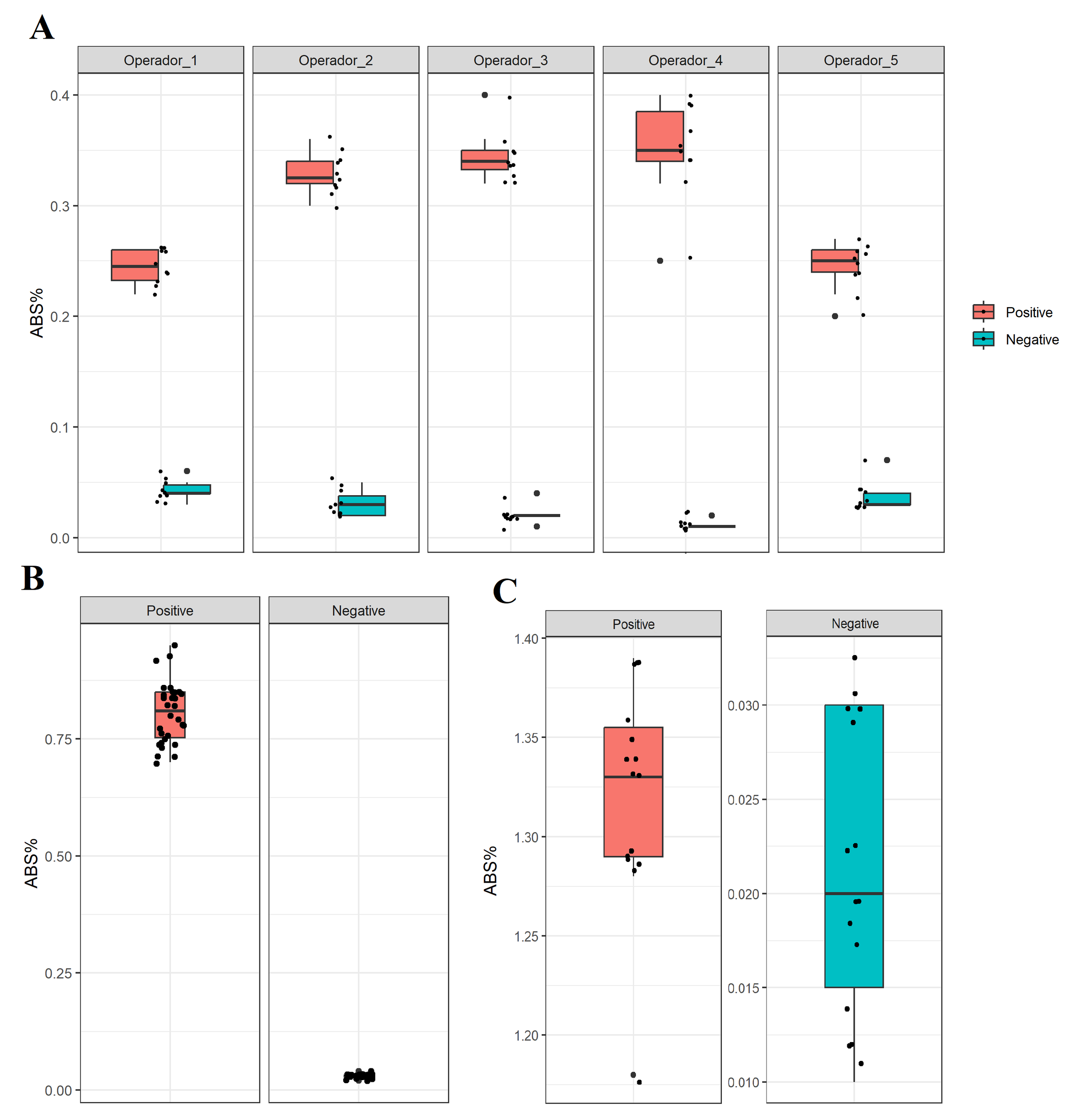

Supplement: S5 Fig — A–reproducibility, B—repeatability, C–homogeneity. ABS%—Percentage of absorbance of the positive standard. (TIF) [file pone.0304268.s006.tif]

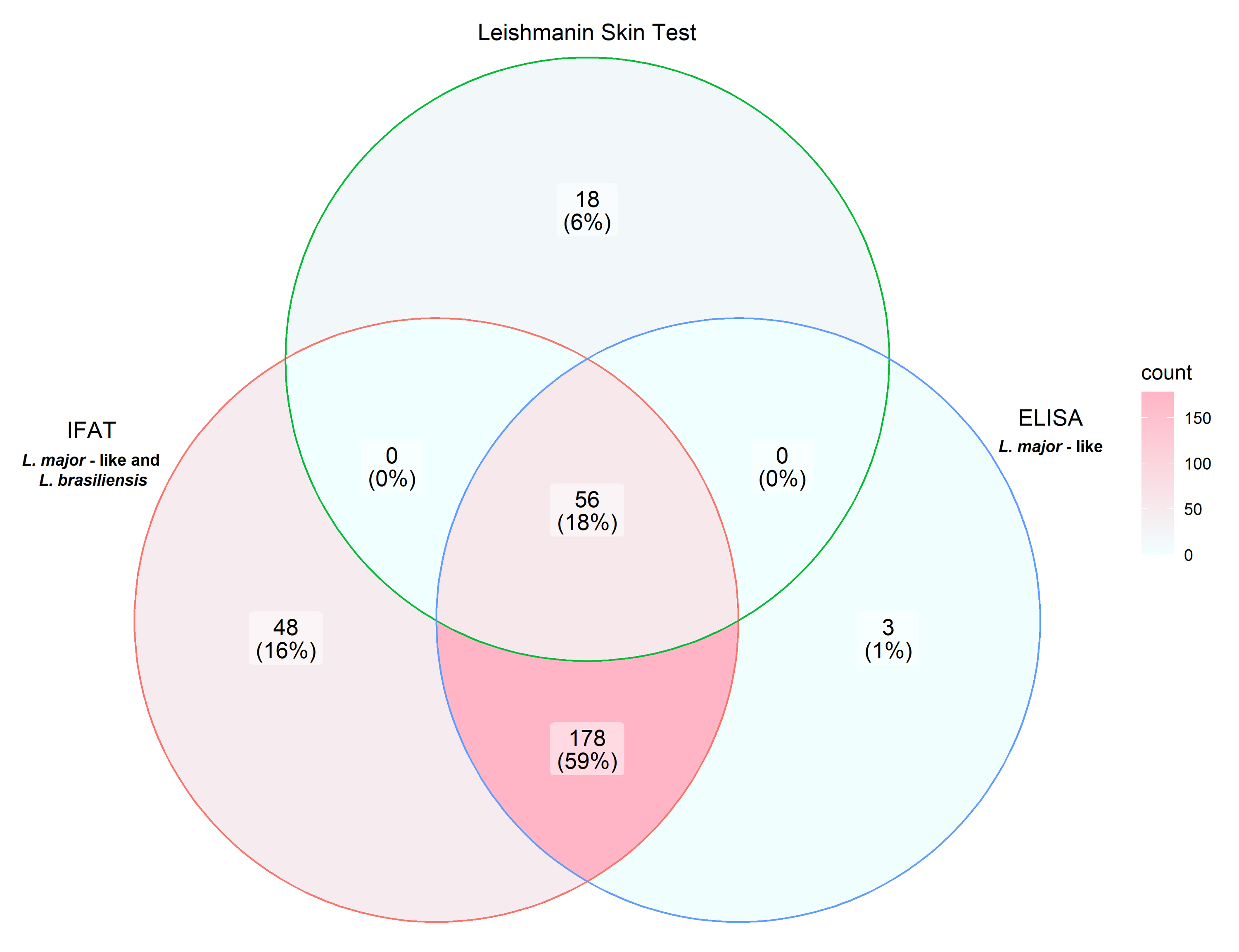

Supplement: S6 Fig — IFAT–Indirect Immunofluorescent antibody test; ELISA–enzyme-linked immunosorbent assay; LST–leishmanin skin test. (TIF) [file pone.0304268.s007.tif]

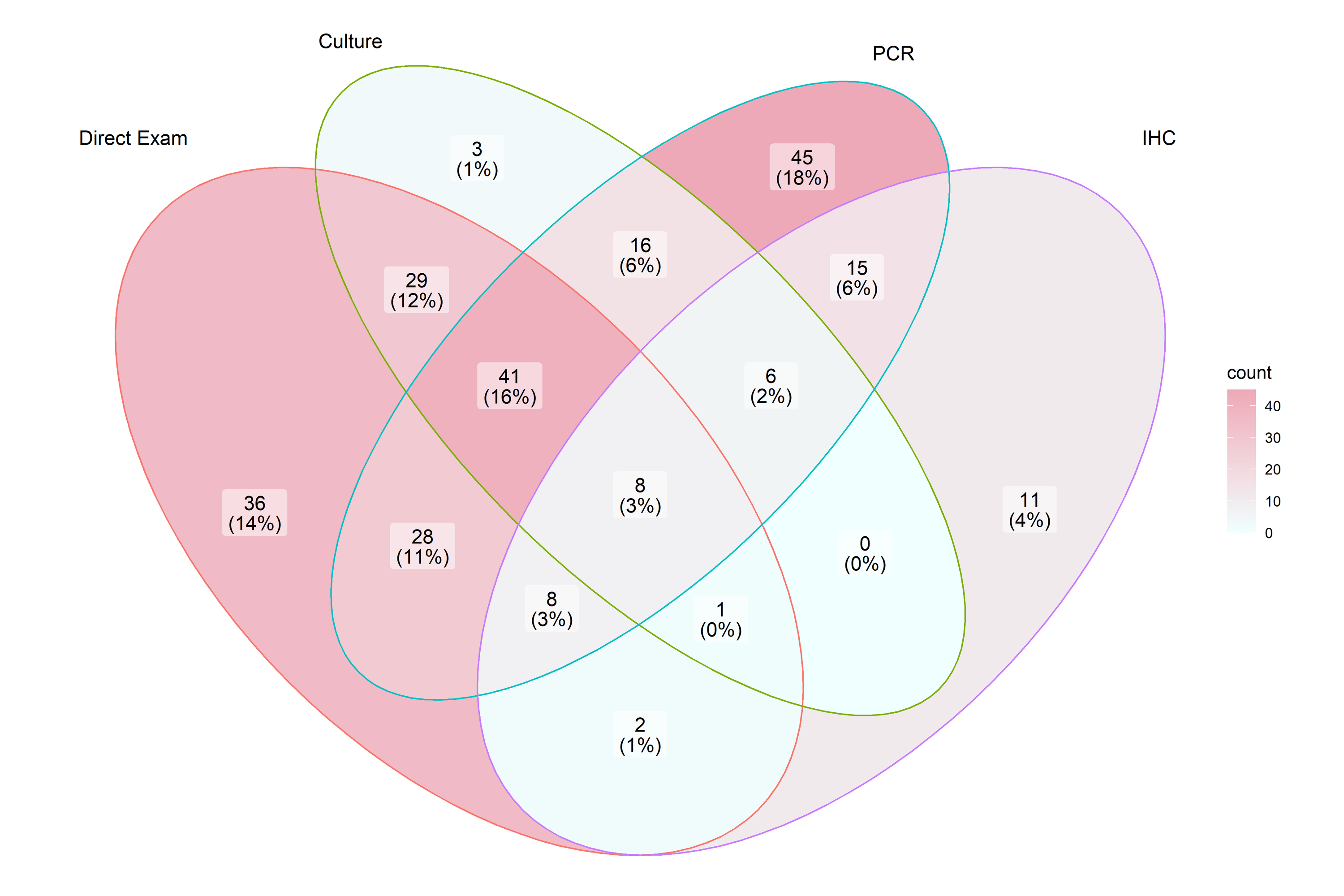

Supplement: S7 Fig — IHC–Immunohistochemistry; PCR–polymerase chain reaction. (TIF) [file pone.0304268.s008.tif]

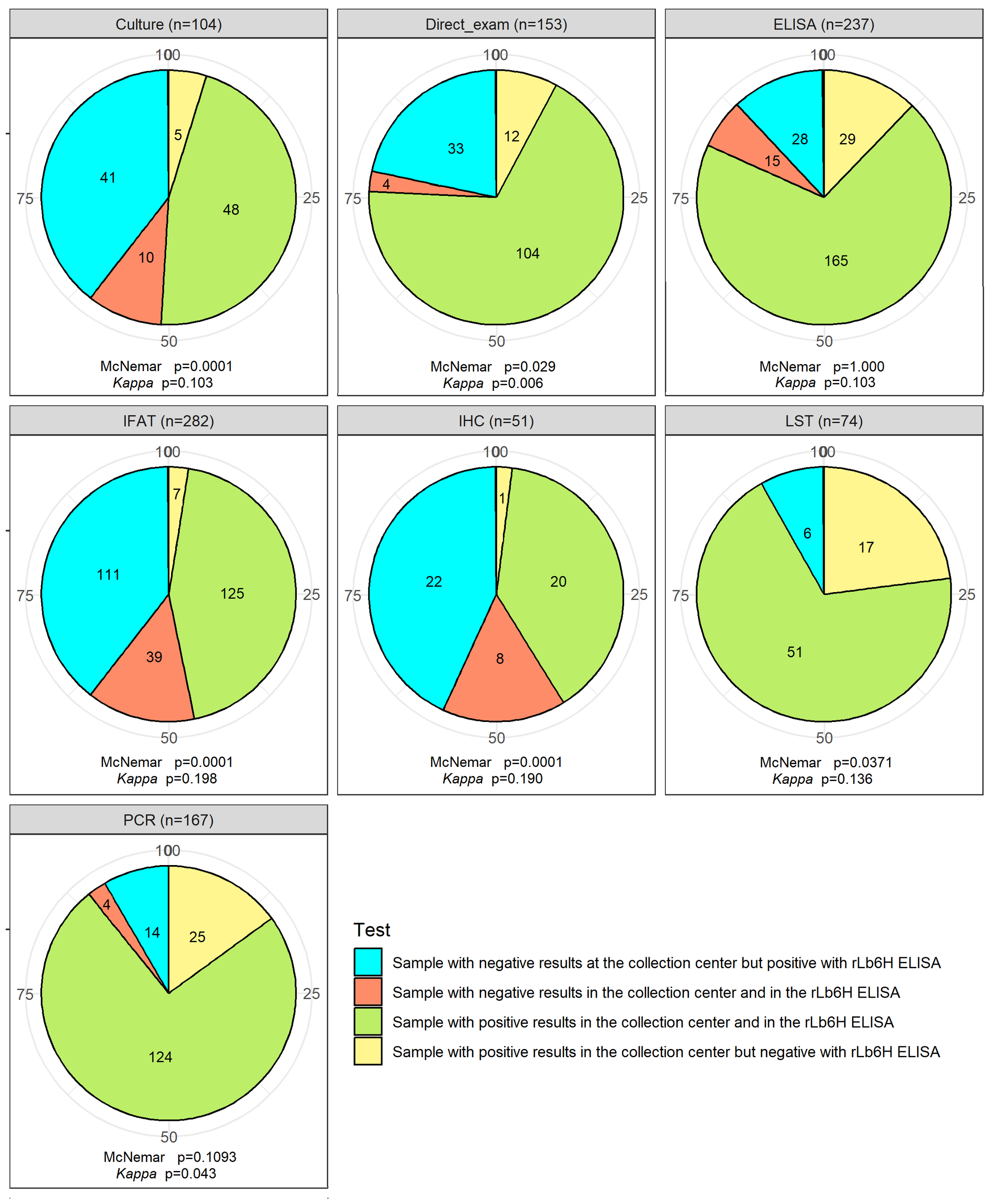

Supplement: S8 Fig — IFAT–immunofluorescent antibody test with L. major-like (Sao Paulo) and L. braziliensis (Manaus); IHC–immunohistochemistry; ELISA–enzyme-linked immunosorbent assay with L. major-like; PCR–polymerase chain reaction; LST–leishmanin skin test. (TIF) [file pone.0304268.s009.tif]
