# Supplementary material for: ELISA with recombinant antigen Lb6H validated for the diagnosis of American tegumentary leishmaniasis
Source: PLoS One. 2024 Jun 5;19(6):e0304268. doi: 10.1371/journal.pone.0304268 (PMC11152253; doi:10.1371/journal.pone.0304268)
Supplement: S1 Table — 1 –Mean optical densities (OD); 2—SD Standard deviation; 3—CV Coefficient of variation; 4 –P samples from patients with American Tegumentary Leishmaniasis (N = 1); 5 –N samples of healthy individuals (N = 1). (PDF) [file pone.0304268.s010.pdf]

S1 Table - Coefficient of variation obtained in the complementary study of the rLb6H-ELISA.

	Homogeneity	Repeatability	Reproducibility
		**Positive**	
	**P**	**P**	**P**
**Mean[1]**	0.50	0.81	0.30
**SD[2]**	0.04	0.07	0.05
**CV[3]**	8.12	8.20	17.97
		**Negative**	
	**N**	**N**	**N**
**Mean[1]**	0.02	0.03	0.03
**SD[2]**	0.01	0.00	0.01
**CV[3]**	34.98	13.88	49.96

1 – Mean optical densities (OD); 2 - SD Standard deviation; 3 - CV Coefficient of variation; 4 – P samples from patients with ATL (N=1); 5 – N samples of healthy individuals (N=1)
