# Supplementary material for: ELISA with recombinant antigen Lb6H validated for the diagnosis of American tegumentary leishmaniasis
Source: PLoS One. 2024 Jun 5;19(6):e0304268. doi: 10.1371/journal.pone.0304268 (PMC11152253; doi:10.1371/journal.pone.0304268)
Supplement: S1 Raw images — (PDF) [file pone.0304268.s012.pdf]

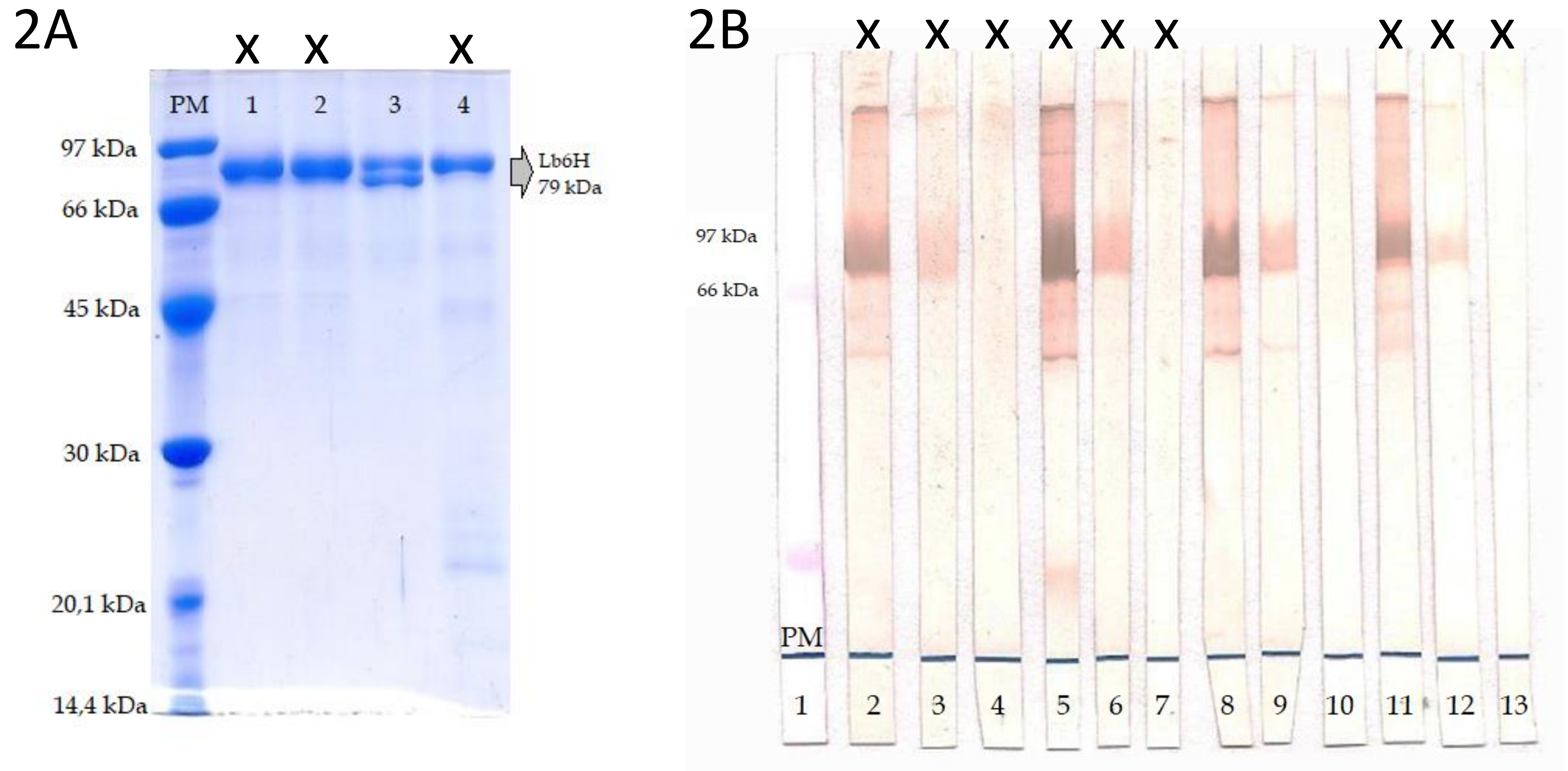

Fig 2. Evaluation of rLb6H antigen. 2A. Electrophoretic profile of rLb6H protein on SDS-PAGE, applying 3µg/mL of protein per well. 2B. Immunoblotting reactivity profile of rLb6H. 1- Molecular weight control stained with Ponceau S. 8 - High titer leishmaniasis positive control. 9 – Low titer leishmaniasis positive control. 10 - Negative control
