# Supplementary material for: ELISA with recombinant antigen Lb6H validated for the diagnosis of American tegumentary leishmaniasis
Source: PLoS One. 2024 Jun 5;19(6):e0304268. doi: 10.1371/journal.pone.0304268 (PMC11152253; doi:10.1371/journal.pone.0304268)
Supplement: Attachment — Submitted filename: Response_to_Reviewers.docx [file pone.0304268.s013.docx]

Dear Editor and reviewers,

Thank you for carefully analyzing our manuscript and for your comments and suggestions. We will reply to your answers and suggestions in the following paragraphs.

Academic editor comments:

Journal requirements:

1. Please ensure that your manuscript meets PLOS ONE's style requirements, including those for file naming. The PLOS ONE style templates can be found at https://journals.plos.org/plosone/s/file?id=wjVg/PLOSOne_formatting_sample_main_body.pdf and https://journals.plos.org/plosone/s/file?id=ba62/PLOSOne_formatting_sample_title_authors_affiliations.pdf

**Answer**: We followed the style PLOS ONE style templates.

2. We note that the grant information you provided in the ‘Funding Information’ and ‘Financial Disclosure’ sections does not match.

**Answer:** We corrected the information. Grant numbers of the awards are provided.

3. PLOS ONE now requires that authors provide the original uncropped and unadjusted images underlying all blot or gel results reported in a submission’s figures or Supporting Information files. When you submit your revised manuscript, please ensure that your figures adhere fully to these guidelines and provide the original underlying images for all blot or gel data reported in your submission. See the following link for instructions on providing the original image data: https://journals.plos.org/plosone/s/figures#loc-original-images-for-blots-and-gels.

**Answer**: We created a PDF containing the original blot and gel images, named “S1_raw_images,” and uploaded it as a Supporting Information file. In the cover letter, we noted that the original images are in the Supporting Information file.

4. Please amend your authorship list in your manuscript file to include authors Dr. Ruth Tamara Valencia-Portillo, Dr. José Angelo Lauletta Lindoso, and Dr. Beatriz Julieta Celeste.

**Answer:** We included the authors.

5. We note that [Figure 1] in your submission contains [map/satellite] images which may be copyrighted.

**Answer:** The map in Figure 1 was generated through RStudio, version 4.3.2 using the geobr package from rstudio.com, and cited in the References.

6. Please review your reference list to ensure that it is complete and correct. If you have cited papers that have been retracted, please include the rationale for doing so in the manuscript text, or remove these references and replace them with relevant current references. Any changes to the reference list should be mentioned in the rebuttal letter that accompanies your revised manuscript. If you need to cite a retracted article, indicate the article’s retracted status in the References list and also include a citation and full reference for the retraction notice.

**Answer:** References were checked and reviewed. Changes were mentioned in the rebuttal letter.

Reviewers' comments:

Reviewer #1: The article consists of the validation of an ELISA using the recombinant protein of an hsp of *L. braziliensis*. The results indicate that the usefulness of this ELISA test is promising. The article is very well written and the results adequately support the conclusions.

**Answer:** Thank you for carefully analyzing our manuscript and for your comments and suggestions.

1. Regarding Lb6H, reviewing the bibliography I must understand that it is the insoluble purification of rLbhsp83a, however, the reference is not clear. It is suggested that this be detailed.

**Answer:** The rLb6H was obtained from the insoluble fraction in the purification process, as cited in Sato's paper (DOI: 10.1128/JCM.01904-16). IDRI provided this protein, and Malcolm Scott Duthie, Jeffery Guderian, and Steven Reed also provided information about it. Please see lines 186-191.

2. Furthermore, it is suggested that the differences that exist between ATL cases with respect to the sample collection center be discussed.

**Answer:** We accepted your suggestion and discussed the differences between ATL cases. Please see the lines 492-499.

Reviewer #2: This is an interesting study with use of serum samples from American tegumentary leishmaniasis patients, health individuals and individuals with other diseases from several endemic and non-endemic areas in Brazil, aiming at evaluating the performance of rLb6H-ELISA as a diagnostic tool. Some issues must be addressed by the authors especially regarding the clarity of the titles/legends in the figures and tables.

**Answer:** Thank you for carefully analyzing our manuscript and for your comments and suggestions.

1. Figures and tables must be clearly presented and be self-explained, so one is not required to go through the text in order to search for explanations of what is stated. In Table 1, it is not clear what “Panel 1” and “Panel 2” mean. Please briefly define in the title or the legend of Table 1 the meaning of “Panel 1” and “Panel 2”. Similarly, in Table 4 add in the title the total number of ATL patients. In Table 5, explain in the legend the meaning of Sp and 95% CI, and in Table 6 explain in the legend the meaning of 95% CI.

**Answer:** We accepted your suggestion to make the tables self-explained.

2. On page 7, lines 137-138: “This study did not include minors.” Table 1 regarding Panel 1 shows age range of 70 patients with ATL from 9 to 84 years, therefore including minors (from Belém-PA and from Recife-PE). Rephrase or withdraw the sentence.

**Answer:** We withdrew the sentence.

3. Page 15, line 323, add “titer”: “(…) the high leishmaniasis (…)” = “(…) the high titer leishmaniasis (…). Also in line 324: “(…) the reaction with the low leishmaniasis (…)” = “(…) the reaction with the low titer leishmaniasis (…)”

**Answer:** We included “titer” where suggested. Please see the lines 323-324.

4. Page 24, lines 510-513: “The excellent performance of rLb6H-ELISA in patients with CL should be highlighted since the diagnosis is more difficult in this form because there are few parasites in the lesion.” In the context of discussing the best performance of the test in CL (86.4% sensitivity) when compared with ML (76.7% sensitivity), this statement implies that lesions in CL have fewer parasites than those of ML, which frequently is not true. Number of parasites depends on the species of the parasite and clinical and immunological factors from the host, like duration of the disease, presence of disseminated or diffuse leishmaniasis, or comorbidities like HIV coinfection. Consider rephrasing the sentence.

**Answer**: We rephrased the sentence and excluded the wrong information. Please see the lines 502-505.

5. Page 26, line 574: Please change “Wirchowian leprosy” to “multibacillary leprosy”.

**Answer:** Thank you for the correction. Please see the line 562.

6. Legend of S1 Figure: Please put Leishmania in Italics.

**Answer:** Thank you for the correction.

Reviewer #3: The manuscript about ELISA-Lb6H for diagnosing American Tegumentary Leishmaniasis (ATL) is interesting. The results obtained indicate that this method has the potential to be used as a complementary test in diagnosis of ATL. It is also important to highlight the use of samples from different regions of Brazil and three neighboring countries; and cases of ATL caused by different species of Leishmania.

**Answer:** Thank you for carefully analyzing our manuscript and for your comments and suggestions.

1. Use of "leishmaniases" and "leishmaniasis". Eg, lines 45, 73 and 76. Standardizes the use of "leishmaniasis".

**Answer**: In line 71, we used Leishmaniases (plural) because we referred to VL and TL forms. When we referred to TL, we standardized Leishmaniasis (singular).

2. S1 dataset: The 21 samples from Recife were identified using Schizodeme. To identify Leishmania species, this method uses parasites, generally isolated by culture. However, for some samples, the authors indicated as "miss information" or negative for cultive and other tests used. How was this identification made?

**Answer:** An aspiration puncture of the lesion was performed for parasite identification, and the material was separated into two parts. One part was inoculated into culture, and the other part into hamsters. In the spreadsheet, the "culture" column refers to the result of direct culture. When bacteria or fungi sometimes contaminated the collected material, isolating the parasite was impossible. In the "species" column, there is the result of the characterization for schizodemas obtained from the parasites recovered from the hamster inoculated and sacrificed after three months or from the culture when it was positive.

3. The authors need to improve the discussion on the use of the composite reference standard (CRS) in their study. In line 585 they mentioned this limitation, but it needs to be discussed further. Composite reference standards have been advocated in diagnostic accuracy studies in the absence of a perfect reference standard. As the number of component tests increases, sensitivity of this CRS increases at the expense specificity, unless all tests have perfect specificity. Therefore, such a CRS can lead to significantly biased accuracy estimates of the index test. The bias depends on disease prevalence and accuracy of the CRS. Some tests used in the CRS of the present study do not have perfect specificity (eg. IFAT and ELISA). I suggest the following reference on this topic: "Schiller et al., 2015. Bias due to composite reference standards in diagnostic accuracy studies. Statistic in medicine. DOI: 10.1002/sim.6803".

**Answer:** We accepted your suggestion and discussed the limitations of using CRS. We also thank you for suggesting this interesting reference. Please see the lines 492-499.

4. Furthermore, it is necessary to improve the discussion on evaluating the clinical specificity of the ELISA-Lb6H assay. It is important to include in the text the need to evaluate more serum samples from patients with symptoms compatible with CL or ML (eg. skin ulcer or ulcer on the oral and/or nasal mucosa), but with a differential diagnosis that excludes ATL.

**Answer:** We included a sentence about the lack of samples of cancer patients with skin or oral/nasal mucosa ulcers. Please see the lines 562-571.

Reviewer #4: The manuscript by Velencia-Portillo et al., aimed to carry out a large-scale evaluation of the use of a bacterially expressed, recombinant protein from *Leishmania braziliensis* for the serological diagnosis of tegumentary leishmaniasis. It builds on previous groups from the same group and others which first assessed the potential use of the same protein for the serological diagnosis of TL. The paper is scientifically sound and is able to evaluate a large number of sera from different sources and also from individuals afflicted by a number of diseases, in order to evaluate cross-reactivity to other diseases. Overall, it strongly supports the use of recombinant proteins for the serological diagnosis of tegumentary leishmaniasis, through ELISA assays, something that has been already clearly stablished for the visceral leishmaniasis, but which has been questioned by some regarding its use for the tegumentary form of the disease. I do find, however, that the manuscript needs a much more detailed description of the antigen it is investigating and why it would be better than others currently assessed for the same purpose. The focus on an antigen that lacks any apparent specificity for tegumentary leishmaniasis should be more clearly explained. Some mention to the serological methods currently used for the visceral leishmaniasis, various commercially available, should also be included.

**Answer:** Thank you for carefully analyzing our manuscript and for your comments and suggestions.

1. The manuscript needs a very thorough revision of the text. The overall organization needs improvement. I find strange, for instance, the large number of paragraphs with single sentences in the Introduction and Discussion. Also, the written English seems in many cases inadequate with many examples where it needs improvement.

**Answer:** We accepted your suggestion and unified many sentences in one paragraph.

2. I could not find any specific details regarding the recombinant protein used, either in the current manuscript or in the cited references. At least a summary of what it is and what is published regarding it should be mentioned and cited adequately.

**Answer:** We accepted your suggestion and summarized details about the recombinant protein and the references. More details about the production of the protein are at <https://doi.org/10.1128/JCM.01904-16>. Please see the lines 186-191.

3. I find odd that the authors do not investigate or detail the specificity of the recombinant protein for the different leishmaniasis. There is a mention of it cross-reacting with visceral leishmaniasis, but it is not detailed in any clear form. If this issue has been investigated or is described elsewhere, it has to be detailed. If not, the reasons why also need to be told. If their protein cannot discriminate between different forms of leishmaniasis, this has to be explicitly stated in the title, abstract in the text. For example, the authors can state that their protein can “confirm the diagnosis of leishmaniasis in individuals with suspected ATL”, they cannot confirm that it is indeed ATL, since a person with visceral leishmaniasis would have a positive result as well.

**Answer:** A previous study (https://doi.org/10.1128/JCM.01904-16) mentioned a cross-reactivity of rLb6H with VL samples using a different batch of the Lb6H antigen. We carried out a test with 45 VL samples, and these results were added to the article text. The purpose of the rLb6H ELISA is to determine the diagnosis of tegumentary leishmaniasis in suspect cases, considering epidemiological and clinical data. However, the reactivity observed with VL does not compromise the use of the antigen in diagnosing ATL, as both forms usually have different symptoms. Please see the lines 516-519.

4. In my view, Tables 1 and 2 and Figure 1 should be included in a first section of the Results, with some of the adjoining text, since a relevant part of the manuscript was devoted to recruiting the various sera groups.

**Answer:** We accepted your suggestion and moved Tables 1 and 2 and Fig 1 to Results.

5. Figure 1 – If the results shown here haven’t been shown before in the previous manuscripts describing the rLb6H recombinant protein, this has to be stated here explaining the need for these experiments at this stage. If similar results have been shown before, there is no need to show them again. Once more, the references to the previous work have to be clearly stated.

**Answer:** Figure 1 is a map showing the “Geographic distribution of 343 American tegumentary leishmaniasis patients (panel 2) according to the likely site of infection”. If you are referring to Figure 2, the results were not presented in previous manuscripts.

6. Figures 5 and 6 show basically the same result. In my view only one or the other should be shown. The other can be left as supplementary.

**Answer:** We accepted your suggestion, and Figure 5 was moved to Supporting Information.

7. Methods – Many paragraphs with single sentences only. In many cases these can be united into a single paragraph.

**Answer:** We accepted your suggestion and unified many sentences in one paragraph.

8. Lines 83-86 – There is not need for such a detailed description of the distribution of ATL cases in Brazil and the reference in Portuguese is not adequate. Reference “3” would also be inadequate for the same reasons in my view.

**Answer:** We accepted your suggestion and excluded the detailed description of the distribution of ATL cases in Brazil.

9. Line 88 – Define “Etiological diagnostic methods”

**Answer:** We accepted your suggestion and defined Etiological diagnostic methods. Please see the lines 83-84.

10. Lines 90-91 – There is no reference to support the following statement “Regarding molecular methods, standardization between laboratories is underway, and their use is still restricted in reference and research centers”.

**Answer:** We accepted your suggestion and added the reference. Please see the line 86.

11. Line 329 – The total amount of protein loaded on the gel should be stated, not just the concentration used (“3μg/mL”).

**Answer:** We accepted your suggestion and stated the amount of protein loaded on the gel. Please see the line 196.

12. Line 340 – Clarify what is the unity of the “cut-off point”, whether is the OD value or else.

**Answer:** The cut-off was calculated by the ROC curve using the absorbance of each sample divided by the absorbance of the positive reference control and multiplied by 100. The cut-off point is a quotient between the absorbances and has no unit. Please see the lines 224-231.

13. Lines 362-365 – Figure S5 comes after Figure S1. Confirm if this is OK.

**Answer:** It is OK.

14. Lines 378-379 – I would expect that the differences in sensitivity observed between FMT-HVD (93.7%) and HCFMUSP (75.6%) would be significant. Please confirm that.

**Answer:** If we had compared 93.7% and 75.6% using Fisher's exact test, we would have achieved a significant difference (p=0.0144). However, we compared all centers, used the chi-square test, and found no difference (p=0.1377).

15. Lines 417-420 – The results showing a greater cross-reactivity with malaria, paracoccidioidomycosis and tuberculosis is noteworthy when the cross-reactivity with Chagas diseases is much lower. The authors should do a better job at discussing why they think this has happened and considering the homology in sequence for the protein between different organisms.

**Answer**: We accepted your suggestion and discussed the homology queries. Please see the lines 551-556.

16. Line 511 – I would not call the performance “excellent”. Please rephrase and tone down.

**Answer:** We accepted your suggestion and rephrased the sentence. Please see the line 573.

17. Lines 479-506 – Please summarize and avoid discussing generalities regarding diagnostic methods and references in Portuguese.

**Answer:** We accepted your suggestion and excluded generalities.

18. Lines 555-595 – Lots of unnecessary text and information. Please summarize it all in one or two paragraphs. Please tone down the potential use of rLb6H-ELISA as an alternative test as it is, due to the substantial amount of cross-reactivity with other diseases. It is a valid work but as an alternative test some improvement is required.

**Answer:** We accepted your suggestion and excluded some paragraphs.
